# Do Spoken Vocabulary and Gestural Production Distinguish Children with Transient Language Delay from Children Who Will Show Developmental Language Disorder? A Pilot Study

**DOI:** 10.3390/ijerph19073822

**Published:** 2022-03-23

**Authors:** Pasquale Rinaldi, Arianna Bello, Francesca Romana Lasorsa, Maria Cristina Caselli

**Affiliations:** 1 Institute of Cognitive Sciences and Technologies, National Research Council, Via Nomentana, 56, 00161 Rome, Italy; cristina.caselli@istc.cnr.it; 2 Department of Education, Roma Tre University, Via Castro Pretorio, 20, 00185 Rome, Italy; arianna.bello@uniroma3.it; 3 Asl Roma 1 TSMREE 13, Via Domenico Silveri, 8, 00165 Rome, Italy; francesca.lasorsa@aslroma1.it

**Keywords:** gesture, language delay, developmental language disorder, picture naming game, MacArthur–Bates Communicative Development Inventory, representational gesture, temporal synchrony, semantic synchrony

## Abstract

The literature on the role of gestures in children with language delay (LD) is partial and controversial. The present study explores gestural production and modality of expression in children with LD and semantic and temporal relationships between gestures and words in gesture + word combinations. Thirty-three children participated (mean age, 26 months), who were recruited through a screening programme for LD. Cognitive skills, lexical abilities, and the use of spontaneous gestures in a naming task were evaluated when the children were 32 months old. When the children were 78 months old, their parents were interviewed to collect information about an eventual diagnosis of developmental language disorder (DLD). According to these data, the children fell into three groups: children with typical development (*n* = 13), children with LD who did not show DLD (transient LD; *n* = 9), and children with LD who showed DLD (*n* = 11). No significant differences emerged between the three groups for cognitive and lexical skills (comprehension and production), for number of gestures spontaneously produced, and for the sematic relationships between gestures and words. Differences emerged in the modality of expression, where children with transient LD produced more unimodal gestural utterances than typical-development children, and in the temporal relationships between gestures and words, where the children who would show DLD provided more frequent representational gestures before the spoken answer than typical-development children. We suggest a different function for gestures in children with T-LD, who used representational gestures to replace the spoken word they were not yet able to produce, and in children with LD-DLD, who used representational gestures to access spoken words.

## 1. Introduction

We explore here the lexical skills, gestural production, and relationship between gestures and words in 3-year-old children with language delay (LD) and with typical language development (TD). There is a large consensus that LD refers to children aged 18 to 35 months who, in the absence of any neurological, sensorial, or cognitive deficits, are slow to develop expressive language (i.e., expressive vocabulary scores ≤10th percentile and/or lack of multiword combinations at 30 months) [1,2,3]. The prevalence of LD depends on the criteria, tools, and age considered, although it is estimated to be close to 20% of children at 2 years of age, and around 10% of children at 3 years of age [4,5,6,7]. About 45% of children with LD receive a diagnosis of developmental language disorder (DLD) from 4 years of age onwards [7,8]. The remaining children recover their linguistic skills by 3 years of age (children with transient LD) or by 4 years of age (children with persistent LD).

Developmental language disorder emerges in the course of development [9] and appears to be associated with linguistic deficits at preschool age and to interfere with the formal learning/education processes at school age [10]. There is consensus in the literature that LD can be associated with weaknesses in several linguistic and non-linguistic aspects. Ref. [7] reported differences between children with LD aged 18–24 months and TD peers for lexical comprehension, gesture + word combinations, and word combinations. Ref. [6] reported that children with LD at 29 months of age were less likely to use pointing gestures, verbal imitation, and symbolic play and were more likely to produce unintelligible words, compared to children of the normative sample of the tool used in the study (i.e., Italian MacArthur–Bates Communicative Development Inventory (MB-CDI)).

Children with LD are a heterogeneous group, according to the severity of the expressive LD and/or the presence of weaknesses in other communicative and linguistic measures as well as in cognitive development measures. Ref. [11] identified three different communicative-linguistic and cognitive profiles for children with LD: (i) children with severe expressive delay, associated with weak comprehension, weak communication engagement, and low cognitive scores; (ii) children with moderate expressive language, associated with strong comprehension, weak communication engagement, and low cognitive scores; and (iii) children with less severe expressive LD compared to the previous two groups. In a more recent longitudinal study, [8] observed children with LD at three ages: 28, 37 and 48 months. They distinguished three groups of children: (i) children with mild delay in lexical and grammar development, but with normal comprehension, who catch up to their peers by 3 years of age (late bloomers); (ii) children with early expressive LD, still persistent at 3 years of age, but with an early good verbal comprehension, with language recovery at around 4 years of age (slow learners); and (iii) children with severe expressive lexical and grammar delay, as well as weaknesses in early syntactic comprehension. Children in this third group were more likely to show DLD.

In a predictive study, [12] reported that none of the linguistic measures they collected when children were 2 years old could predicted DLD at 4 years of age. Among the measures collected when these children were 3 years old, both the verbal quotient (assessed using the Wechsler Preschool and Primary Scale of Intelligence-III) and the mean length of utterances predicted DLD at 4 years of age. In addition, they showed that at 3 years of age, a number of biological and environmental risk factors have crucial roles in predicting DLD at 4 years of age, even if there was wide heterogeneity in the risk factors for these children (e.g., cognitive development, family history of language/learning problems, prematurity, directive language stimulation style, level of parental education).

However, a recent meta-analysis by [13] highlighted that receptive and expressive lexical skills can explain a small (but significant) amount of the variance in the outcome of children with LD.

Several studies on language acquisition have demonstrated close relationships between gesture and language in both typically developing children and children with developmental disorders (e.g., [14]). Some authors have suggested that gestures are manifestations of the simulated actions and perceptions that underlie thinking [15,16]. Gestures and speech are thus considered as part of the same cognitive and communicative system [17,18]. The same sensorimotor processes that underlie speech are the basis for spontaneous gesture production, and meanings might be conveyed as a synthetic visible depiction, through the hands, in gesturing, when the children have specific deficits in their speech and language skills [19].

Studies that have specifically aimed to investigate the use of gestures and their relationships with co-occurring speech in children with LD have provided conflicting findings, and there is some debate as to how frequently, for what purpose, and how accurately children with LD produce gestures. As evident in the following review of studies, the conflicting findings are probably due to differences in the ages of the participants involved (from 2–3 years to 7 years); the type of the communicative gestures analysed (e.g., only deictic gestures, only symbolic gestures, both of these together); the context in which data on gestures produced by children were collected (e.g., spontaneous gestures, elicited gestures, reproduction of a model); the cognitive and linguistic complexity of the task (e.g., description, narrative, naming); and clinical profiles of the children included.

In their seminal studies, refs. [20,21] explored gestural production within the context of a familiar script by children with LD between 18 and 33 months of age. Children with transient LD produced significantly more communicative gestures, as both deictic gestures (e.g., pointing) and representational/symbolic gestures (e.g., making a drinking gesture without a cup in hand or with a block), compared to children with persistent LD. Ref. [22] explored the use of gestures in two groups of 2.7- to 6.1-year-old children: those they defined as “children with language impairment” and TD children. Children were observed using a picture narration task, and their fine and gross motor competences and linguistic verbal abilities were assessed. The gestures were classified according to the semantic relationship with co-occurring speech: reinforcing, disambiguate, and add. No mean group differences were seen for either measure. They also reported that with respect to TD children, the children with language impairment used gestures at a higher rate and produced greater proportions of gestures without speech and gestures that added novel information to co-occurring speech. Poorer skills in language production were related to more frequent gesture production only in the group of children with language impairment. These latter results are different from those of [23] for older children with specific language impairment (i.e., DLD in current terminology; [7,9,10]), where a specific task was designed to elicit gesture production from pictures. Botting and colleagues showed no differences in the number of gestures produced between TD children and children with DLD. In addition, they showed a positive and significant correlation between expressive language and gesture production skills only in the children with DLD. A subsequent study [24] examined gesture production and gesture comprehension in children with receptive-expressive LD and in children with expressive LD, aged 25 to 35 months. The Communication and Symbolic Behaviour Scales [25] and the Early Socio-Cognitive Battery [26] were used to assess gesture production and gesture comprehension, respectively. Children with expressive LD understood and produced a greater number of gestures than children with receptive-expressive LD. In addition, children with better receptive language were more likely to have higher scores for gesture production and comprehension, and children with higher scores in gesture comprehension were more likely to have higher scores for gesture production. In contrast, expressive language was not significantly related to gesture production or comprehension. In a longitudinal study, [27] reported that first communicative gestures at 15 months of age (i.e., deictic gestures, conventional gestures) did not directly contribute to the risk of language impairment at 3 and 4.5 years of age. The effects of gestures on the risk of language impairment were mediated by vocabulary production (but not vocabulary comprehension).

Other studies have analysed the role and function of gestures in (older) children whose LD resulted in DLD; however, the results of these studies are controversial and do not allow definitive interpretation. Ref. [28] studied spontaneous gesture production in a naming task (the same task used in the present study) in children with specific language impairment (i.e., DLD) aged 3.6 to 5.6 years, and in two groups of TD children, where one group was matched for chronological age (CATD) and the other for linguistic abilities (LATD), according to the mean length of utterance. Deictic gestures were the gestures most frequently produced by children in all of the groups. Children with DLD did not differ from the LATD children in terms of the number of deictic and representative gestures produced, while both of these groups produced higher numbers of deictic and representative gestures than the CATD children. In addition, they showed that children with DLD and LATD children produced significantly higher numbers of gestures, which reinforced the correct information conveyed in co-occurring speech, compared to the CATD children. These results are in agreement with the hypothesis that gesture could facilitate the retrieval of the spoken word and/or could support the conceptual multimodal packaging of information before it is coded into a linguistic form for speech by facilitating its spatial-motor encoding and its organisation for speaking [29,30].

In this pilot study, we explored lexical skills, gesture production, and the relationship between gestures and words in three groups of 3-year-old children, as TD children and two groups with LD: one with transient LD (T-LD) and the other with subsequent diagnosis of DLD at 4 to 5 years of age (LD-DLD). The specific aims were to investigate similarities and differences between these three groups of children in spoken lexical abilities using a structured naming test: the picture naming game (PiNG), considering both comprehension and production; in the number and type of gestures spontaneously produced during the naming test; in the modality of expression used by the children (i.e., only spoken, only gestural, and gesture + word); and in the semantic and temporal relationships between gestures and co-occurring words in bimodal answers. We hypothesized that the children with T-LD (i.e., who will not show DLD at 3 years of age) would have the following:

(i.) similar numbers of correct answers in lexical comprehension compared to TD children, but higher than LD-DLD children. This hypothesis relies on the literature that demonstrates that children with LD who also have weaknesses in comprehension skills are more likely to have DLD (e.g., [8]).
(ii.) lower numbers of correct answers in lexical production than TD children, but higher than LD-DLD children, and lower numbers of items without a spoken answer and unintelligible answers than LD-DLD children. These hypotheses rely on the literature on children with LD, and in particular on findings on the relationships between the severity of lexical delay and the probability of resultant DLD (e.g., [8,11]) and on the relationships between expressive vocabulary size and types of errors in a naming task [31,32,33].
(iii.) higher numbers of both deictic and representative gestures (produced alone or in word combinations) than the TD and LD-DLD children. This issue is still controversial, as the great majority of studies have been conducted with older children. Studies on young children with LD have shown that children with expressive LD (and thus less likely to result in DLD) produced higher numbers of gestures than children with receptive-expressive LD (and were thus more likely to result in DLD) (e.g., [20,21,24,33]).
(iv.) the same semantic relationships between gestures and co-occurring words in bimodal answers with respect to the TD and LD-DLD children, and the same temporal relationships as LD-DLD children, with a higher number of gestures produced before the words, compared to TD children, as gestures might facilitate the process of lexical retrieval in children with language difficulties. No studies have analysed the temporal relationships between gestures and co-occurring words in children with LD. One study on school-age children reported no significant differences among children with DLD and two groups of TD children (one matched for chronological age, the other for language development) in gesture–speech synchrony, as the Group × Relationship interaction was not significant [34].

We believe that this pilot study may contribute to defining the communicative and linguistic profiles of 3-year-old children with LD who will recover their linguistic skills by 4 years of age and those of children who have a higher risk of DLD.

## 2. Methods

### 2.1. Participants and Recruitment

The participants of this pilot study were selected from children who participated in a language screening programme for LD when they were 24 to 30 months old. The language screening programme was carried out in 12 kindergartens in Rome and involved 227 monolingual children.

The screening tool used was the (Italian) parental Words and Sentences MB-CDI short-form questionnaire [35]. Children with an expressive vocabulary ≤10th percentile were considered as children with LD, which was seen for 60 of these children (26.4%). At 3–6 months from the screening programme, all these children were invited to participate in the second phase of the study, which included administration of the Italian MB-CDI complete form and direct cognitive and linguistic assessment. Thirty-two of the 60 families with children with LD (53%) agreed to participate. The remaining 28 families did not agree to participate. Twenty-nine of the 32 children (91%) who took part in the second step of the study were confirmed with LD (≤10th percentile in the vocabulary size for MB-CDI complete form). Among these, 20 families gave permission to video record the sessions of direct assessment and gave their telephone numbers and agreed to be contacted in the future, according to the aims of the study. When the children were 6 to 7 years old, the parents were contacted for a telephone interview, in which they were asked for information about an eventual diagnosis of DLD received according to the results of clinical assessment, made by a team including a neuropsychiatrist, a neuropsychologist, and a speech therapist, when the children were 4 to 5 years old. According to the answers of the parents, 11 children received a diagnosis of DLD, while the remaining nine children did not receive such a diagnosis.

In addition, 20 TD children at the screening phase (i.e., expressive vocabulary >10th percentile) were invited to participate in the second phase of the study as the control group. All of these children were confirmed as showing typical language development, as they were above the 10th percentile in the Words and Sentences MB-CDI complete form. The families of 13 of these children gave permission to video record the sessions of direct assessment and gave their telephone numbers and agreed to be contacted in the future, according to the aims of the study. The families of all 13 of these children confirmed during the telephone interview that these children did not receive any diagnosis of DLD when they were 4 to 5 years old.

To summarize, the TD children were those with TD at the screening phase and at the direct assessment, and with no diagnosis of DLD at 4 to 5 years of age; the T-LD children showed LD at the screening phase and at the direct assessment, although no diagnosis of DLD at 4 to 5 years of age; and the LD-DLD children showed LD at the screening phase and at the direct assessment, plus diagnosis of DLD at 4 to 5 years of age.

The demographic characteristics of these three groups of children and the results of the statistical analysis for differences between the groups in terms of chronological age, gender, cognitive development (IQ), familiarity with LD and DLD, and maternal education level are shown in Table 1. None of the children had sensory or neurological deficits or intellectual disabilities. None of the children were preterm or twins. All of the children were monolingual Italian and had been exposed since birth to only Italian. No significant differences emerged among these three groups for any of the internal or individual and external or environmental factors considered.

### 2.2. Instruments

#### 2.2.1. Words and Sentences MB-CDI Short Form

At the screening phase, the parents of the children were asked to fill in the Italian version of the Words and Sentences MB-CDI short form questionnaire [35]. This tool was validated on 816 Italian children aged 18 to 36 months and showed a high concurrent validity (r = 0.92) with the Words and Sentences MB-CDI complete form [36]. It was also shown to be suitable for screening projects for the identification of children with LD [6]. It contains four sections, but for the purposes of the present study, only the data for the first section are reported: the vocabulary checklist. The vocabulary checklist estimates the child’s expressive vocabulary size and includes a list of 100 words. Parents were asked to indicate the words that were produced by their child. At the end of the questionnaire, the additional information collected was the family educational level and the child’s medical history and familiarity with language and/or learning disorders.

#### 2.2.2. Words and Sentences MB-CDI Complete Form

During the direct assessment, the parents of the children were asked to fill in the Italian version of the Words and Sentences MB-CDI complete form [35]. This tool was validated on 572 Italian children aged 18 to 36 months [35,36], and its use is recommended for research and in the clinical setting, because it is informative both in terms of quality and quantity of information [35]. For the purposes of the present study, only the data for the vocabulary checklist are reported. The vocabulary checklist estimates the child’s expressive vocabulary size and includes a list of 670 words. The parents were asked to indicate the words that were produced by their child.

#### 2.2.3. The Picture Naming Game

The PiNG was administered to the children during the direct assessment. It is a task used to assess lexical comprehension and production, and it has been validated on 388 Italian children aged 19 to 37 months [32]. This tool includes four subtests, with 20 items each: noun comprehension, noun production, predicate comprehension, and predicate production. The nouns subtest represents objects and tools, whereas the predicates subtests represent actions, attributes, and locative adverbs. To analyse lexical comprehension, three pictures were presented for each item (lexical target, semantically related distractor, and semantically unrelated distractor). The tester pronounced the word referring to the picture, and the child was asked to touch the corresponding picture. To analyse the lexical production, the pictures were presented one by one, and the child was asked to name the picture. Only one answer was coded for each item (the better one, in the case of multiple answers of the child). The order of picture presentation within each subtest was fixed.

#### 2.2.4. Bayley Scales of Infant and Toddler Development

The Bayley Scales of Infant and Toddler Development, third edition [37,38], was administered to the children during the direct assessment, to exclude children with intellectual disabilities. A composite score was calculated for each child (M = 100, SD = 15).

#### 2.2.5. Parental Interview

The parents of the children were contacted by telephone when the children were 6 to 7 years old. The structured interview included the following closed-ended questions (yes/no): Q.1. Did your child undergo a clinical assessment for neurodevelopmental or behavioural disorder? Q.2. Did your child receive a diagnosis of language disorders when (s)he was 4–5 years old, as a result of clinical assessment? Q.3. Did your child receive a diagnosis of other neurodevelopmental or behavioural disorder? Q.4. Did your child receive any intervention for language disorder? The time requested for each interview was about 5 min.

### 2.3. Procedure

The data in the present pilot study were collected in three phases. The first one was the screening, when the children were 24 to 30 months old, in which the percentile of expressive vocabulary size was computed through the Italian version of the Words and Sentences MB-CDI short form, which was filled in by the parents. The second phase was the direct assessment, when the children were 30 to 36 months old. Their parents were asked to fill in the Italian version of the Words and Sentences MB-CDI complete form. The children were individually tested at their home or in our laboratory, where the PiNG task and the Bayley Scales of Infant and Toddler Development were administered. This session was entirely video recorded, as our interest was in spontaneous gesture production during the PiNG task. The video-recorded administration of the PiNG task allowed us to examine the gestural production, which was the target of this study. Verbal and gestural productions were transcribed using the ELAN software [39]. The third phase took place when the children were 4 to 5 years old. The parents underwent a structured telephone interview.

The parents provided informed written consent for participation in the study, which included permission to video record the assessments and to be contacted by telephone in the future. The study met the ethical guidelines for human subject protection, including adherence to the legal requirements of the country (Declaration of Helsinki), and it received formal approval by the local research Ethical Committee of the Roma Tre University (date of approval: 18 March 2020).

### 2.4. Coding

#### 2.4.1. MB-CDI Short Form and Complete Form: Lexical Vocabulary

For the MB-CDIs, the percentiles for word production were computed on the basis of the number of words produced by the children and their ages, following the data reported by [36].

#### 2.4.2. PiNG: Spoken Answer Accuracy

The answers from the children during the PiNG task were coded according to the coding procedures of the validation study [32]. For the comprehension subtests, only the first answer was analysed. For the production subtests, if the child did not provide the target answer on the first attempt, a second opportunity was given (without correcting the child). In these cases, only the “best answer” was considered.

For the comprehension subtests, the children’s answers were considered as “correct” when they showed, touched, or pointed to the picture corresponding to the target word pronounced by the experimenter. For the production subtests, the children’s answers were considered as “correct” when the child provided the target word corresponding to the picture. Phonologically altered forms were also considered as correct answers. No-spoken answers (i.e., an answer without a spoken element) and unintelligible answers (i.e., verbal forms for which it was not possible to recognize any correspondence to a word) were also calculated.

As not all of the children received all of the items of the production subtests (due to fatigue or to evident difficulties in answering the test), the proportions (%) of each type of answer (correct answers, no-spoken answers, and unintelligible answers) were calculated for the number of items administered for each subtest.

#### 2.4.3. PiNG: Modality of Expression and Types of Gestures

For the modality of expression, the answers provided during the production subtests were coded as unimodal spoken answers, unimodal gestural answers, and bimodal answers, according to [40]. Unimodal spoken answers (e.g., the child says ‘table’ without producing any gesture) included answers that contained only words, with no pointing or representational gestures. Unimodal gestural answers (e.g., the child runs his/her hand through the hair with spread fingers for ‘comb’, without producing any word) included answers that contained only pointing or representational gesture(s), with no words. Bimodal answers (e.g., the child produces ‘lion’ and holds his/her hands as claws in a menacing way near the head) included answers that contained word(s) and pointing or representative gesture(s). Unimodal spoken answers and bimodal answers included correct, incorrect, and unintelligible answers.

Representative gestures included both manual gestures and all visible representative actions that involved postures, body movements, and facial expressions that were spontaneously produced by the children [41]. Only one gesture per item was considered. For answers where a child produced a pointing gesture and a representative gesture, the representative gesture was considered. For answers where a child provided more than one gesture, only the gesture close to the “best” spoken answer was considered.

As not all of the children received the same number of items in the subtest of noun and predicate production, gestural production was calculated as a proportion (%) of the number of items administered.

#### 2.4.4. Semantic and Temporal Relationship between Gesture and Speech

The semantic and temporal relationships between gestures and co-occurring words were analysed only for representative gestures produced in bimodal answers. For the semantic relationship, the procedure suggested by [42] was followed. The gesture + word combinations were classified as “productions with equivalent meaning” when the meanings expressed by the verbal answer and the gesture were the same (e.g., the child wraps fingers around an imaginary comb, moving near the head, as for combing his/her hair and says ‘comb’), and as “productions with supplementary meanings” when the two answers provided different meanings (e.g., the child turns her/his finger to her/his cheek while saying ‘banana’).

For the temporal relationships, the onset of the gesture and the onset of the pronunciation of the word were considered. Gestures were classified as (i) “produced before”, if the onset of the gesture occurred before the onset of the word; (ii) “produced during”, if the onset of the gesture and the onset of the word occurred at the same time; and (iii) “produced after”, if the onset of the gesture occurred after the onset of the word. In addition, the proportion (%) of each of these types of temporal relationship out of the total representative gestures produced was computed.

### 2.5. Reliability

To calculate the agreement between the two raters, for agreement on the presence/absence of a gesture, on the type of gesture produced (i.e., deictic gesture or representative gesture), and on the semantic relationship between the representative gestures and co-occurring words (i.e., equivalent gesture or supplementary gesture), we used Cohen’s kappa [43]. For agreement on the type of answer produced by the children (i.e., unimodal spoken answer, unimodal gestural answer, or bimodal answer) and on the temporal relationships between the representative gestures and co-occurring words (i.e., gesture produced before, during, or after co-occurring word), we used Fleiss’s kappa [44].

The video recordings of the productions were provided by seven participants (21%) and were independently coded by two raters. We first calculated the agreement on the presence/absence of the gesture (i.e., the child produced a gesture or the child did not produce a gesture) and on the type of answer produced by the children (i.e., unimodal spoken answer, unimodal gestural answer, or bimodal answer). The proportion of the overall agreement on the presence/absence of a gesture was 94.0% (K = 0.87), and the proportion of overall agreement on the type of answer was 88.4% (K = 0.81). The agreement on the type of gesture produced (i.e., deictic gesture or representative gesture) was calculated only for the items in which a gesture was produced, where the proportion of overall agreement was 97.7% (K = 0.95). The agreement on the semantic and temporal relationships between the representative gestures and co-occurring words (i.e., equivalent gesture, supplementary gesture, gesture produced before, during, or after co-occurring word) were calculated only for the items in which a representative gesture was produced. The proportion of overall agreement for the semantic relationship was 89.7% (K = 0.79), and for the temporal relationship, it was 93.8% (K = 0.64). According to [45], the values of kappa obtained can be interpreted as indices of substantial agreement (kappa 0.61 to 0.80) and almost perfect agreement (kappa 0.81 to 1.00). The two coders discussed any disagreements while watching the relevant video recordings again and then came to an agreement.

### 2.6. Statistical Analysis

The significance for the comparisons between groups in terms of gender, family history of language and/or learning disorders, and mother’s educational level, and the differences between the three groups of children for the numbers of children who were <10th percentile in the PiNG subtests, were determined using Chi-squared tests.

The comparisons between the groups in terms of IQ and chronological age at the screening phase, the direct assessment phase, and the parental interview phase, and the differences between the three groups in the percentiles obtained for the MB-CDIs, in the scores obtained for the PiNG tests, and in gesture production were explored using separate one-way ANOVAs. Significant differences were determined using post hoc analysis, with Bonferroni correction for multiple comparisons.

Relationships between expressive vocabulary size and scores obtained at the PiNG task were explored using Pearson’s correlations.

A *p* value of <0.05 was considered to be statistically significant.

## 3. Results

### 3.1. Spoken Lexical Abilities

#### 3.1.1. Indirect Measures: MB-CDI Percentiles

Table 2 gives the descriptive data for the three groups of children and the results of the statistical analyses for the differences between the groups for vocabulary size for the MB-CDI short form and complete form.

The children in the three groups showed significantly different percentiles of expressive vocabulary as estimated using both the MB-CDI short form at the screening phase and the MB-CDI complete form at the later assessment phase. Post hoc analysis with Bonferroni correction for multiple comparisons showed that, as expected, the TD children had higher percentiles than both the T-LD and LD-DLD children, but no significant differences were found between the T-LD and LD-DLD children.

#### 3.1.2. Direct Measures: PiNG Task Subtests

Table 3 gives the numbers and proportions of children <10th percentile in each subtest of the PiNG task, i.e., noun comprehension, predicate comprehension, noun production, and predicate production. The normative data of that tool are provided as percentile scores for each of the subtests, and it is not possible to compute a general score for the whole task. Table 3 also provides the numbers of correct answers in the comprehension subtests and the proportions of correct answers of items without spoken answers (no spoken answers) and of unintelligible answers for the production subtests, out of the numbers of items administered. The mean numbers of items administered in the production subtests (out of 40 items) changed according to the different groups of children. They were 40, 30 and 32 for TD, T-LD and LD-DLD, respectively.

For the subtests of the PiNG task, the three groups significantly differed for the proportions of children who fell below the 10th percentile. Post hoc analysis with Bonferroni correction for multiple comparisons showed that the proportions for the TD children were significantly lower than for the T-LD and LD-DLD children, with no significant differences between the T-LD and LD-DLD children.

In the comprehension subtests, the three groups of children significantly differed for the numbers of correct answers. Post hoc analysis with Bonferroni correction for multiple comparisons showed that the TD children provided higher numbers of correct answers than the T-LD and LD-DLD children, with no significant differences between the T-LD and LD-DLD children.

In the production subtests, the three groups of children significantly differed in the proportions of correct answers out of the number of items administered. Post hoc analysis with Bonferroni correction for multiple comparisons showed that the TD children provided greater proportions of correct answers than the T-LD and LD-DLD children, with no significant differences between the T-LD and LD-DLD children.

Again, for the production subtests, the three groups of children also differed in the proportions of items without a spoken answer and with unintelligible answers. Post hoc analysis with Bonferroni correction for multiple comparisons showed that the TD children had fewer items without a spoken answer than the T-LD children. However, the LD-DLD children did not differ from the other two groups here, although they tended to provide higher proportions of unintelligible answers than the TD children (*p* = 0.064). The T-LD children did not significantly differ from the TD and LD-DLD children for the proportion of unintelligible answers.

### 3.2. Relationships between Expressive Vocabulary Size and Scores in the PiNG Task

To explore the relationship between expressive vocabulary size and some scores obtained in the PiNG task, we ran correlational analyses within each group of children, between their expressive vocabulary size as assessed through the MB-CDI and some scores obtained in the PiNG task, as (i) the number of correct answers, (ii) the number of items without a spoken answer, and (iii) the number of unintelligible answers. All of three groups of children showed positive and significant correlations between their expressive vocabulary size and the number of correct answers (TD children: r = 0.659; *p* = 0.014; T-LD children: r = 0.721; *p* = 0.028; LD-DLD children: r = 0.665; *p* = 0.026). The correlations between expressive vocabulary size and number of items without a spoken answer were not significant for any of the three groups of children, while the correlation between expressive vocabulary size and number of unintelligible answers was negative and significant only for the LD-DLD children (r = −0.673; *p* = 0.048); i.e., the greater their vocabulary size, the fewer their numbers of unintelligible answers.

### 3.3. Gestural Production: Types of Gestures and Modality of Expression

The mean number of gestures produced by children in the production subtests changed according to the different group of children. It was 20.08 (16.92 deictic gestures; 3.15 representative gestures), 14.33 (8.11 deictic gestures; 6.22 representative gestures), and 11.82 (6.45 deictic gestures; 5.36 representative gestures) for TD, T-LD, and LD-DLD, respectively. Table 4 gives the proportions of items for which the children produced gestures, out of the number of items administered in the production subtests, as well as the modality of expression used by the children in answering the items of the tests.

In the production subtests, the three groups of children did not differ in the proportions of gestures produced (out of the number of items administered), of deictic gestures produced, and of representative gestures produced.

In the production subtests, for the modality used by the children in answering the items, the three groups did not differ in the proportions of spoken unimodal answers and of bimodal (gesture + word) answers. In contrast, for the proportions of gestural unimodal answers, post hoc analysis with Bonferroni correction for multiple comparisons showed that the T-LD children provided higher proportions of gestural unimodal answers than the TD children. However, the LD-DLD children did not significantly differ from the TD and T-LD children for gestural unimodal answers. As for gestural unimodal answers, no significant difference emerged among the three groups in the proportion of answers with a representative gesture (88%, 73%, and 78% for TD, T-LD, and LD-DLD groups, respectively), as well as in the proportion of answers with a deictic gesture (12%, 27%, and 22% for TD, T-LD, and LD-DLD groups, respectively). With respect to bimodal answers, no significant difference emerged among the three groups in the proportion of answers with a representative gesture (16%, 35%, and 37% for TD, T-LD, and LD-DLD groups, respectively), as well as in the proportion of answers with a deictic gesture (84%, 65%, and 63% for TD, T-LD, and LD-DLD groups, respectively). All groups produced a higher proportion of representative gestures in gestural unimodal answers and, conversely, produced a higher proportion of deictic gestures in bimodal answers.

Taking into account only the answers provided by the children in which a deictic gesture was involved (i.e., gestural unimodal answers and bimodal answers with deictic gestures), a significant difference emerged. The T-LD children provided a lower proportion of bimodal productions with a deictic gesture (and thus a higher proportion of gestural unimodal answers with a deictic gesture) than the TD children. The LD-DLD children did not differ from the TD and T-LD children in the types of answers (i.e., gestural unimodal or bimodal) in which deictic gestures were produced.

Taking into account only the answers provided by the children in which a representative gesture was involved (i.e., gestural unimodal answers and bimodal answers with representative gestures), a further significant difference emerged. The T-LD children provided a lower proportion of bimodal productions with a representative gesture (and thus a higher proportion of gestural unimodal answers with a representative gesture) than the TD children. Again, the LD-DLD children did not differ from the TD and T-LD children in the types of answer (i.e., gestural unimodal or bimodal) in which representative gestures were produced.

Taking these last two significant results together, it can be seen that the T-LD children used gestures (either deictic or representative) without words (i.e., gestural unimodal productions) more frequently than the TD children, who in turn used gestures (either deictic or representative) more frequently together with words (i.e., bimodal productions).

### 3.4. Semantic and Temporal Relationships in Bimodal Productions

To investigate the semantic and temporal relationships in bimodal productions (gesture + word), only answers in which a representative gesture was involved were considered. The bimodal productions (representative gesture + word) were considered as “equivalent” when they had the same meaning and were considered as “supplementary” when the two meanings were different. They were also classified as “before”, “during”, or “after” according to the timing of the gesture production (i.e., “before”, “during”, or “after” the verbal response, respectively). Table 5 gives the semantic relationships between the gestures and words in bimodal answers with a representative gesture.

The three groups of children did not differ in the proportions of bimodal productions in which the semantic relationship between gesture and word was equivalent or supplementary.

Table 6 gives the temporal relationships between gestures and words in bimodal answers with a representative gesture.

The three groups of children significantly differed in the proportions of representative gestures produced before the spoken answer. Post hoc analysis with Bonferroni correction for multiple comparisons showed that the LD-DLD children provided representative gestures before the spoken answer more frequently than the TD children. The T-LD children did not differ from the TD and LD-DLD children. Furthermore, the three groups of children did not differ in the proportions of representative gestures produced either during or after the spoken answer.

## 4. Discussion

In this pilot study, we explored lexical skills, gestural production, and the relationship between gestures and words in three groups of 3-year-old children: TD children (i.e., >10th percentile for lexical production at 24 to 30 months old, no diagnosis of DLD at 4 to 5 years old); T-LD children (i.e., <10th percentile for lexical production at 24 to 30 months old, no diagnosis of DLD at 4 to 5 years old); and LD-DLD children (i.e., <10th percentile for lexical production at 24 to 30 months old, plus diagnosis of DLD at 4 to 5 years old). The specific aims were to preliminarily explore similarities and differences among these three groups of children in terms of their spoken lexical abilities, using the Italian MB-CDI filled in by their parents and through direct observation using the PiNG task, as well as in terms of spontaneous gesture production.

For the PiNG task, the following variables were considered: lexical comprehension and lexical production; number and type of gestures spontaneously produced; modality of expression used by the children (only spoken productions, only gestural productions, or gesture + word productions); and semantic and temporal relationships between gestures and co-occurring words in bimodal answers.

### 4.1. Spoken Lexical Abilities: Indirect and Direct Measures

As expected, the TD children had significantly higher percentiles of expressive vocabulary (estimated with the MB-CDIs) than the T-LD and LD-DLD children. However, no significant differences were seen between these last two groups of children with LD.

With respect to the results obtained using the PiNG task, for receptive vocabulary, we hypothesized that the T-LD children at 3 years of age would have lexical comprehension skills similar to those of the TD children, but higher than the LD-DLD children. The results show that comprehension did not differ between the two groups of children with LD (i.e., the T-LD and LD-DLD children). This was probably because of the small number of participants in the sample. In addition, the PiNG task assesses the comprehension of lexical elements, which is quite different with respect to grammar comprehension, also in terms of cognitive load, as assessed in other studies that have highlighted that the outcome of DLD can be predicted by comprehension skills (e.g., [8]).

For lexical production, we hypothesized that at 3 years of age, the T-LD children would have lower numbers of correct spoken answers than the TD children, but higher than the LD-DLD children, plus a lower number of items without a spoken answer and unintelligible answers than the LD-DLD children. As expected, the TD children had higher scores than both the T-LD and the LD-DLD children, although no significant difference emerged between the T-LD children and the LD-DLD children for the proportions who fell below the 10th percentile in the subtests of the PiNG task and in the proportions of correct answers in the production subtests. These results were relatively unexpected, as many studies have shown that expressive vocabulary size can predict the risk of a DLD outcome in children with LD (e.g., [8,11]); however, a meta-analysis by [13] highlighted that receptive and expressive lexical skills can explain a small (but significant) amount of the variance in the outcome of children with LD.

The T-LD children provided higher numbers of answers without a spoken element than the TD children (but not different from LD-DLD children), and the LD-DLD children provided higher numbers of unintelligible answers than the TD children (but not different from T-LD children). Thus, none of these measures was able to differentiate at 3 years of age the children who would recover their LD (i.e., the T-LD children) from the children with LD who would show DLD (i.e., the LD-DLD children).

Several studies have already reported high rates of no spoken answers and/or answers with phonological simplifications and unintelligible answers in children with LD assessed through structured tasks (for review, see [46]). Ref. [47] reported high individual variability among children with LD in terms of no answers and answers with phonological errors. They used a non-word repetition task and a naming task, through which they identified different profiles. Some children used a no answer strategy (no responders, in their terminology), while other children attempted to produce the target word, although with phonological errors. In addition, they reported that the subgroup of children who used the highest proportion of no responses had the lowest expressive vocabulary size, while the subgroup of children who attempted to produce the target word, even with phonological errors, had larger expressive vocabulary [47].

This type of analysis is not adequate for our sample, as our groups of children with LD did not differ in terms of expressive vocabulary size. Nonetheless, to explore this issue, we ran correlational analyses within each group of children, between their expressive vocabulary size as assessed through the MB-CDI and some scores obtained in the PiNG task. All three groups of children showed positive and significant correlations between their expressive vocabulary size and the number of correct answers. The correlations between expressive vocabulary size and number of items without a spoken answer were not significant for any of the three groups of children, while the correlation between expressive vocabulary size and number of unintelligible answers was negative and significant only for the LD-DLD children; i.e., the greater their vocabulary size, the fewer their numbers of unintelligible answers.

This is also in agreement with the results of previous studies on children with typical and atypical language development, as well as in children at high risk for language delay [31,33,48]. These studies used the same naming task as used in the present study and showed a relationship between expressive vocabulary size and types of answers.

### 4.2. Gestural Production: Type of Gesture and Modality of Expression

According to some evidence from the literature (e.g., [24,49,50]), we hypothesized that children at 3 years of age with LD who would not show DLD (i.e., the T-LD children) would produce higher numbers of gestures (produced alone or in gesture + word combinations) than the TD and LD-DLD children. However, our results did not confirm this hypothesis. Indeed, the three groups of children who participated in the current study did not differ in the proportions of deictic and representative gestures produced (out of the number of items administered) in the production subtests.

For the modality used by the children in answering the items in the production subtests, the three groups did not differ in the proportions of spoken unimodal answers and in the proportions of bimodal (gesture + word) answers. In contrast, the three groups of children differed in the proportions of gestural unimodal answers: the T-LD children provided higher proportions of gestural unimodal answers than the TD children, while the LD-DLD children did not significantly differ from the TD and T-LD children. In addition, considering the higher proportions of items without spoken answers provided by the T-LD children, this result is in agreement with the theories that have suggested that gestures can sustain the meaning acquisition process, by helping children to express meanings that they already know, but for which they have either not yet mastered the corresponding spoken label or have not yet had a stable representation [29].

Our preliminary findings are consistent with those of [22] for a sample of preschoolers with language impairment compared with their TD peers in a picture narration task, which suggested that gestures might have compensatory roles when language is delayed or impaired. The analysis of spontaneous gestural productions and of gesture + word combinations is very useful to study the communicative and linguistic profiles of children with LD. However, according to the results of this pilot study, the analysis of spontaneous gestural productions and of gesture + word combinations does not appear to be adequate to distinguish between 3-year-old children with LD who will recover their LD (i.e., the T-LD children) from those who will result in DLD (i.e., the LD-DLD children).

### 4.3. Semantic and Temporal Relationships in Bimodal Productions

For the semantic relationships, we hypothesized that children at 3 years of age with LD who did not show later DLD (i.e., the T-LD children) would have the same semantic relationship between gestures and co-occurring words in bimodal answers with respect to the TD and LD-DLD children. As expected, the three groups of children did not differ in the proportions of bimodal productions in which the semantic relationship between gesture and word was equivalent or supplementary. As these three groups of children were matched for cognitive development, this result suggests that the children in these three groups had similar levels of semantic representation, although different relationships between semantic representations and spoken lexical repertoire.

For the temporal relationship, we hypothesized that both groups of children with LD (i.e., the T-LD and LD-DLD children) would produce more gestures before the spoken word. Our results partly confirmed this hypothesis, as the LD-DLD children provided representative gestures before the spoken answer more frequently than the TD children, although as often as the T-LD children. This lack of difference between these two groups of children with LD is in agreement with the lack of difference in their lexical skills (which were assessed in the same sessions in which gestural productions were evaluated). In addition, it is worth noting that the TD children never produced gestures before the spoken answer; only the children in the two groups with LD did this. From a qualitative perspective, a continuum was indeed seen, from the TD children who never produced gestures before spoken words, to the T-LD children who produced a few gestures before spoken words, to the LD-DLD children who produced gestures before spoken words more frequently. The production of gestures that anticipate the spoken answers in children who still have a limited vocabulary size might be considered as a root for the retrieval of the spoken label, to allow the child to access the link between the meaning (concept) and the spoken lexical label, as suggested by [51].

## 5. Conclusions

Several studies have highlighted changes in the predictors of DLD as a function of age. According to the findings of this pilot study, receptive and expressive vocabulary size, as well as the number of gestures spontaneously produced in a naming task, are not reliable measures to use in the third year of life to distinguish the outcomes of children with LD. The literature on gesture production in children has highlighted that the individual variability is very high and that gesture production can also depend on communicative attitude, temperamental traits, age, and spoken lexical skills. The limited number of children in this pilot study and the high individual variability might have hidden a potential significant relationship between gesture production and outcome. In addition, children with LD were split in two different subgroups (i.e., T-LD and LD-DLD) according to parents’ answers on questions asking for eventual diagnosis of DLD received for their children after a clinical assessment. Further studies should also include a direct evaluation of children to confirm the eventual diagnosis of DLD made by clinicians.

In this pilot study, for the first time, the temporal relationships between spontaneous gestures and co-occurring words were investigated in toddlers. In contrast to TD children, children with LD (and in particular those who will show DLD) produced gestures before the spoken words in bimodal productions. This is in agreement with theories that have suggested that gesture has a prime role in lexical production by facilitating the retrieval of the spoken word when the speaker has weakness in accessing their lexicon. It is also in agreement with a different, but not alternative, theoretical perspective that has suggested that gestures support the conceptual multimodal packaging of information before it is coded into a linguistic form for speech by facilitating its spatial-motor encoding and its organisation for speaking ([30,52]; for a review and discussion on this topic, see [53]). To rephrase [54], children whose language difficulties persist during development are not necessarily those with the most severe initial difficulties and/or those who produce fewer gestures.

The present study does not provide clear evidence on the role of gestures at 3 years of age to distinguish children with LD who will have different developmental outcomes. The limited number of children, as well as the assignment of children in the group of T-LD and LD-DLD using a parental interview, lead us to consider this research as a pilot study. Further studies using different tasks and different observational contexts are necessary to more deeply investigate the relationships between spontaneous gestures and spoken language in children with LD and to understand to what extent gestures before 4 years of age can have predictive roles in distinguishing children who will recover from their LD (i.e., T-LD children) from those who will show DLD (i.e., LD-DLD children).

## Figures and Tables

**Table 1:** Characteristics of the children included in the study.

Characteristic	Detail	Group			Chi^2^/F (df)	*p*
		>TD (*n* = 13)	>T-LD (*n* = 9)	>LD-DLD (*n* = 11)		
Chronological age (months) (mean (SD) (range))	Screening phase	25.46 (0.97) (24–27)	26.00 (2.24) (24–29)	26.73 (1.42) (24–28)	F(2,30), 2.007	0.152
	Direct assessment	32.62 (1.76) (30–36)	32.00 (1.80) (30–34)	36.64 (1.69) (29–35)	F(2,30), 0.419	0.661
	Telephone interview	78.08 (6.56) (68–94)	78.44 (6.27) (71–89)	76.55 (5.92) (68–87)	F(2,30), 0.272	0.763
Gender (*n* (%))	Female	5 (38.5)	3 (33.3)	2 (18.2)	Chi^2^(2), 1.214	0.545
Intelligence quotient (mean (SD) (range))		102.15 (9.19) (82–117)	100.13 (10.05) (90–115)	99.22 (7.21) (90–109)	F(2,27), 0.314	0.733
Family history of language and/or learning disorders, *n* (%)	Yes	4 (30.8)	2 (22.2)	7 (63.6)	Chi^2^(2), 4.224	0.121
Mothers with medium/high educational level (>13 years), *n* (%)	Yes	9 (69.2)	3 (33.3)	6 (54.5)	Chi^2^(2), 2.764	0.251

**Table 2:** Percentiles for the Italian version of the Words and Sentences MB-CDI short form and complete form.

MB-CDI	Group Percentiles (Mean (SD) (Range))			F(df)	*p*
	TD (*n* = 13)	T-LD (*n* = 9)	LD-DLD (*n* = 11)		
Short form	50.54 (19.16) (32–92)	3.78 (3.56) (1–10)	3.09 (2.59) (1–9)	F(2,30), 57.45	<0.0001
Complete Form	48.69 (23.26) (14–85)	2.0 (1.73) (1–5)	3.0 (1.9) (1–6)	F(2,30), 38.41	<0.0001

**Table 3:** Measures from the PiNG task.

Task	Detail	Group			Chi^2^/F (df)	*p*
		TD (*n* = 13)	T-LD (*n* = 9)	LD-DLD (*n* = 11)		
Percentile						
Comprehension subtests (<10th percentile, *n* (%))	Noun	0 (0)	5 (55.6)	4 (36.4)	Chi^2^(2), 8.963	0.011
	Predicate	0 (0)	3 (33.3)	6 (54.6)	Chi^2^(2), 9.167	0.010
Production subtests (<10th percentile, *n* (%))	Noun	2 (15.4)	8 (88.9)	10 (90.9)	Chi^2^(2), 21.758	<0.0001
	Predicate	2 (15.4)	9 (100)	10 (90.9)	Chi^2^(2), 18.381	<0.0001
Type of answers						
Comprehension subtests (mean (SD) (range))	Number of correct answers	35.08 (1.5) (33–38)	27.11 (7.59) (14–33)	28.73 (5.35) (21–37)	F(2,30), 7.898	0.002
Production subtests (mean (SD) (range))	Proportion (%) of correct answers ^a^	63.28 (16.52) (28–83)	16.71 (15.85) (0–50)	22.06 (21.08) (0–65)	F(2,30), 23.349	<0.0001
	Proportion (%) of no-spoken answers ^a^	4.15 (4.69) (0–17)	12.11 (8.33) (5–28)	9.4 (8.49) (0–25)	F(2,29), 3.598	0.040
	Proportion (%) of unintelligible answers ^a^	0.85 (0.9) (0–3)	7.56 (10.6) (0–34)	8.2 (8.08) (0–23)	F(2,29), 3.737	0.036

^a^, out of the relevant number of items administered.

**Table 4:** Measures from the gestural production.

Category	Detail	Group (Mean (SD) [Range])			F (df)	*p*
		TD (*n* = 13)	T-LD (*n* = 9)	LD-DLD (*n* = 11)		
Gesture production						
Production subtests	Proportion (%) of gestural production (deictic and representative) ^a^	50.53 (22.56) (18–83)	43.78 (19.76) (22–80)	40.43 (31.60) (0–100)	F(2,30), 0.498	0.613
	Proportion (%) of deictic gestures ^a^	42.60 (23.33) (5–78)	27.42 (17.15) (8–58)	25.77 (36.31) (0–100)	F(2,30), 1.396	0.263
	Proportion (%) of representative gestures ^a^	7.93 (7.97) (0–23)	16.36 (22.55) (0–65)	14.66 (15.52) (0–50)	F(2,30), 0.947	0.399
Modality of expression						
Spoken unimodal answers	Proportion (%)	45.54 (21.76) (18–82)	30.78 (18.88) (0–59)	53.91 (30.78) (0–100)	F(2,30), 2.233	0.125
Gestural unimodal answers	Proportion (%)	2.38 (4.15) (0–15)	23.28 (31.46) (0–100)	8.55 (9.59) (0–25)	F(2,30), 3.921	0.031
Bimodal answers	Proportion (%)	52.0 (23.21) (19–83)	45.89 (26.01) (0–76)	37.55 (32.40) (0–100)	F(2,30), 0.836	0.443
Type of gesture produced in unimodal and in bimodal answers						
Answers with a deictic gesture	Proportion (%) of unimodal answers ^a^	1.18 (2.94) (0–9)	25.19 (37.64) (0–100)	4.31 (10.02) (0–29)	F(2,27), 3.700	0.038
	Proportion (%) of bimodal answers ^a^	98.82 (2.94) (91–100)	74.81 (37.64) (0–100)	95.69 (10.02) (71–100)	F(2,27), 3.700	0.038
Answers with a representative gesture	Proportion (%) of unimodal answers ^b^	11.33 (14.55) (0–43)	58.33 (35.34) (12–100)	36.8 (38.77) (0–100)	F(2,20), 5.000	0.017
	Proportion (%) of bimodal answers ^b^	88.67 (14.55) (57–100)	41.67 (35.34) (0–88)	63.2 (38.77) (0–100)	F(2,20), 5.000	0.017

^a^, out of the relevant number of answers with a deictic gesture. ^b^, out of the relevant number of answers with a representative gesture.

**Table 5:** Semantic relationships between representative gestures and words in bimodal productions.

Semantic Relationship	Detail	Group (Mean (SD) (Range))			F(2,18)	*p*
		TD (*n* = 13)	T-LD (*n* = 9)	LD-DLD (*n* = 11)		
Productions with equivalent meaning	Proportion (%)	76.63 (28.56) (25–100)	57.48 (41.11) (0–100)	75.82 (19.09) (50–100)	0.766	0.479
Productions with supplementary meaning	Proportion (%)	23.37 (28.56) (0–75)	42.52 (41.11) (0–100)	24.18 (19.09) (0–50)	0.766	0.479

**Table 6:** Temporal relationships between representative gestures and words in bimodal productions.

Temporal Relationship	Detail	Group (Mean (SD) (Range))			F(2,17)	*p*
		TD (*n* = 13)	T-LD (*n* = 9)	LD-DLD (*n* = 11)		
Representative gestures produced before the spoken answer	Proportion (%)	0 (0) (0–0)	3.13 (6.25) (0–13)	8.88 (8.46) (0–20)	5.302	0.016
Representative gestures produced during the spoken answer	Proportion (%)	48.00 (39.32) (0–100)	41.39 (35.02) (26–100)	45.74 (32.74) (0–100)	0.097	0.908
Representative gestures produced after the spoken answer	Proportion (%)	52.00 (39.32) (0–100)	55.48 (33.59) (0–74)	45.37 (37.58) (0–80)	0.046	0.955

## Data Availability

The data presented in this study are available on request from the corresponding author. The data are not publicly available due to privacy.
